# Molecular Mechanism Associated With the Impact of Methane/Oxygen Gas Supply Ratios on Cell Growth of *Methylomicrobium buryatense* 5GB1 Through RNA-Seq

**DOI:** 10.3389/fbioe.2020.00263

**Published:** 2020-04-07

**Authors:** Lizhen Hu, Yongfu Yang, Xin Yan, Tianqing Zhang, Jing Xiang, Zixi Gao, Yunhao Chen, Shihui Yang, Qiang Fei

**Affiliations:** ^1^School of Chemical Engineering and Technology, Xi’an Jiaotong University, Xi’an, China; ^2^State Key Laboratory of Biocatalysis and Enzyme Engineering, Environmental Microbial Technology Center of Hubei Province, and School of Life Sciences, Hubei University, Wuhan, China; ^3^Key Laboratory of Agricultural Environmental Microbiology, Ministry of Agriculture, College of Life Sciences, Nanjing Agricultural University, Nanjing, China; ^4^Shaanxi Key Laboratory of Energy Chemical Process Intensification, Xi’an Jiaotong University, Xi’an, China

**Keywords:** methanotroph, *Methylomicrobium buryatense*, CH_4_/O_2_ gas supply ratio, transcriptomics, methane metabolism, nitrogen fixation

## Abstract

The methane (CH_4_)/oxygen (O_2_) gas supply ratios significantly affect the cell growth and metabolic pathways of aerobic obligate methanotrophs. However, few studies have explored the CH_4_/O_2_ ratios of the inlet gas, especially for the CH_4_ concentrations within the explosion range (5∼15% of CH_4_ in air). This study thoroughly investigated the molecular mechanisms associated with the impact of different CH_4_/O_2_ ratios on cell growth of a model type I methanotroph *Methylomicrobium buryatense* 5GB1 cultured at five different CH_4_/O_2_ supply molar ratios from 0.28 to 5.24, corresponding to CH_4_ content in gas mixture from 5% to 50%, using RNA-Seq transcriptomics approach. In the batch cultivation, the highest growth rate of 0.287 h^–1^ was achieved when the CH_4_/O_2_ supply molar ratio was 0.93 (15% CH_4_ in air), and it is crucial to keep the availability of carbon and oxygen levels balanced for optimal growth. At this ratio, genes related to methane metabolism, phosphate uptake system, and nitrogen fixation were significantly upregulated. The results indicated that the optimal CH_4_/O_2_ ratio prompted cell growth by increasing genes involved in metabolic pathways of carbon, nitrogen and phosphate utilization in *M. buryatense* 5GB1. Our findings provided an effective gas supply strategy for methanotrophs, which could enhance the production of key intermediates and enzymes to improve the performance of bioconversion processes using CH_4_ as the only carbon and energy source. This research also helps identify genes associated with the optimal CH_4_/O_2_ ratio for balancing energy metabolism and carbon flux, which could be candidate targets for future metabolic engineering practice.

## Introduction

As climate change and global warming become severe, much attention has been given to explore more efficient and economical approaches to utilize the GHGs. Methane (CH_4_) as one of the major GHGs has a much greater global warming potential than CO_2_. Currently, more than 50% of the CH_4_ emission is contributed by natural gas from energy extractions and biogas from agricultures and enteric fermentations (Opio et al., 2013). Because of the development of horizontal drilling and hydraulic fracturing techniques, the unconventional natural gas (shale gas and tight gas) is produced at a surplus globally in last decade. The tremendous increase in shale gas also caused a huge amount gas flared annually at extraction sites around the world. Therefore, it is urgent to capture CH_4_ and fulfill the value of this feedstock.

Methanotrophic bacteria are a subset of methylotrophs, which are capable of using CH_4_ as the sole carbon source for cell growth and biosynthesis of metabolites. Thus, the biological conversion of CH_4_ has been gathering more and more interest as a key role in global carbon cycle. So far, significant efforts have been undertaken to utilize CH_4_ as the substrate to produce biofuels and biochemicals by methanotrophs.

*Methylomicrobium buryatense* 5GB1 is a type I methanotroph that assimilates CH_4_ to methanol using methane monooxygenase (pMMO) first, and then metabolizes methanol mainly through RuMP pathway for pyruvate production and cell growth (Fu et al., 2017). Recently, *M. buryatense* 5GB1 has been proved as a promising industrial strain due to its robust growth as well as the established genetics and synthetic biology approaches for strain engineering. A series of genetic constructions and metabolic engineering works of *M. buryatense* 5GB1 have been carried out to enhance the carbon flux for the production of biomass, lipids, lactic acid, fatty acids, etc. (Dong et al., 2017; Fu et al., 2017; Hu et al., 2017; Garg et al., 2018). The highest lipid productivity of 45 mg/L/h along with the growth rate of 0.22 h^–1^ and DCW of 21 g/L has been achieved by knocking out glycogen biosynthesis genes of *M. buryatense* 5GB1 in batch cultures (Fei et al., 2018). The flux balance model of *M. buryatense* 5GB1 also suggests that the maximum carbon conversion efficiency (CCE) can be as high as 60∼70% on molar basis. Garg et al. (2018) have successfully improved the CCE 50 times by using suitable promoters and RBS combinations for lactic acid production by *M. buryatense* 5GB1.

*Methylomicrobium buryatense* 5GB1 is an aerobic obligate methanotroph, and the oxygen supply is an essential factor influencing cell growth, electron transfer, and energy supply. It has been projected that the growth rate is associated with the consumption rates of CH_4_ and O_2_, which may be caused by the differences of NADH production via oxidative phosphorylation. Similar phenomenon has been observed in cultures of *M. buryatense* 5GB1 with two different CH_4_/O_2_ ratios of inlet gas mixtures. It was found that a better growth and gas update rate were obtained in the cultures with CH_4_/O_2_ ratios of 4.4 on mole basis, compared with that of 0.14 (Zhu et al., 2017).

Moreover, transcriptomic studies suggested that the expression of several key genes associated with NADH formation (formaldehyde to carbon dioxide) was changed dramatically under O_2_-limited conditions (Gilman et al., 2015). Akberdin et al. (2018) also demonstrated that *Methylomicrobium alcaliphilum* 20Z cultured under aerobic or micro-aerobic conditions affected the formation of pyruvate and biomass significantly. It has also been shown that *M. buryatense* 5GB1 exhibited a complex metabolism under O_2_ starvation (Gilman et al., 2017). These findings suggest that under different CH_4_/O_2_ gas supply ratios, the expression of certain genes can be regulated between aerobic methanotrophic growth and denitrification phases, which is important for industrial applications of methanotrophs.

Although applying optimal CH_4_/O_2_ ratios of the inlet gas exhibits significant effects on the overall CCE of metabolic pathways related to cell growth, carbon flux, reducing power and energy generation, few studies have thoroughly explored the CH_4_/O_2_ ratios of the inlet gas, especially for the CH_4_ concentrations within the explosion range (5∼15% of CH_4_ in air), which is mainly due to the safety concerns. We have already developed an automatic control system with CH_4_ alarm and cut-off device along with gas mass flow meters to keep operation running smoothly and safely (Fei and Pienkos, 2018). From the standpoint of revealing the influence of the inlet gas ratio of CH_4_/O_2_ on cell growth, nutrients utilization and energy consumption, it is desirable to understand the effects of various inlet gas ratios of CH_4_/O_2_ on methanotrophic metabolism using a lab-scale, safe bioconversion system (Fei and Pienkos, 2018).

To explore the changes at transcriptional levels globally, many researchers have applied transcriptomics to their studies. Recently, Fu et al. (2019) reported significant differences between growth on CH_4_ and methanol by transcriptional analysis in *M. buryatense* 5GB1. Similarly, a comparative transcriptome analysis was utilized in *Methylomonas* sp. DH-1 to explore the roles of secondary metabolite pathways during growth on CH_4_ and methanol (Nguyen et al., 2019). However, there is no systematical research on *M. buryatense* 5GB1 under different CH_4_/O_2_ ratios.

In this study, five different inlet gas ratios of CH_4_/O_2_ (including three ratios within explosion range for the first time) were investigated in methanotrophic cultivation with continuous supply of CH_4_ and O_2_ in bioreactors. Cell growth and key metabolites were measured to analyze the impact of different CH_4_/O_2_ ratios on *M. buryatense* 5GB1, and transcriptomics study was performed to identify genes differentially expressed under these conditions to help elucidate the impact of CH_4_/O_2_ ratio on cell growth and metabolism of *M. buryatense* 5GB1. The effective gas supply ratio identified in this study could help enable the production of key intermediate such as pyruvate, which is an important precursor for various downstream biochemical biosynthesis, and therefore offers a potentially efficient path to produce biochemicals using CH_4_ as the only carbon source by improving nutrient utilization and energy supply.

## Materials and Methods

### Strain and Culture Conditions

*Methylomicrobium buryatense* 5GB1 obtained from Prof. Mary Lidstrom was revived on NMS agar medium for 5 days, while seed culture was prepared in a gas cylinder filled with gas mixture of 20% CH_4_ in air at 30°C. The medium composition of NMS used in batch cultures was mentioned previously (Fei et al., 2018). Aerobic fermentation was carried out in a 3 L fermentation cylinder (BaoXing Bio-engineering Equipment, Shanghai, China) containing 2 L of modified NMS2 (8 g/liter KNO_3_ and 2 × trace elements) medium by mass-flow gas meter controlling mixed gas content with reliable explosion-proof equipment and good ventilated condition. The composition of the gas blends used in this experiment was as follows: 0.28 (#1, 5% CH_4_ in air), 0.58 (#2, 10% CH_4_ in air), 0.93 (#3, 15% CH_4_ in air), 1.31 (#4, 20% CH_4_ in air), and 5.24 (#5, 50% CH_4_ in air).

### Cell Growth and Metabolite Measurements

Optical density of cell was measured at 600 nm with a spectrophotometer (TU-1810, PERSEE, Beijing, China). As for DCW, sampling was performed every 12 h. 10 mL bacterial solution was placed in a 15-mL centrifuge tube each time and centrifuged at 7,000 rpm for 15 min. The supernatant was discarded, and cells were then resuspended with 5 mL deionized water. Centrifugation step was repeated once. Cells were preserved in the −80°C refrigerator for 4 h, which was then dried by a lyophilizer for 36∼48 h before measuring the dry weight of the cells. Statistical analysis was performed in Microsoft Excel 2007, and *P*-values with statistical significance of *P* < 0.05 and *P* < 0.01 were obtained.

Total protein and pyruvate were determined using kits from Beyotime and Suzhou Comin Biotechnology. The total protein was measured using the Bradford Protein Assay Kit (Beyotime, Shanghai, China). Briefly, 1 mL bacterial culture was transferred to the 1.5 mL microcentrifuge tubes for cell lysis. The absorbance values of the samples diluted with water at 595 nm were measured by UV-spectrophotometry (TU-1810, PERSEE, Beijing, China). Pyruvate was measured by Pyruvate Content Determination kit (Suzhou Comin Biotechnology Inc, Suzhou, China) following the manual. The supernatant was removed by centrifugation, and the precipitate was resuspended with extracting solution before cell lysis using the ultrasonic cell breaker (YH-1000Y, Ningbo, China). The procedure also involved in adding a single working reagent for pyruvate determination at 520 nm by UV-spectrophotometry.

### Enzymatic Assays

Particulate methane monooxygenase activity was measured using propene epoxidation assay as described before (Miyaji et al., 2002). The intracellular NADH and NAD^+^ concentrations were measured by the enzyme cycling method (He et al., 2016). Briefly, 1-mL bacterial liquid was transferred to the 1.5-mL microcentrifuge tubes for NADH or NAD^+^ extraction. After centrifugation, cell pellets were resuspended with 300 μL 0.2 M NaOH for NADH quantification or 300 μL 0.2 M HCl for NAD^+^ determination. The samples are bathed in 50°C for 10 min and immediately placed in iced water. The cell lysate was neutralized by adding the same volume of HCl or NaOH. The supernatants were stored at −20°C and used to detect the NADH and NAD^+^ activity after centrifuging at 15,000 rpm for 5 min. The solution (total volume was 0.6 mL) contained 0.1 mL of each of followings: 1.0 M Bicine buffer (pH 8.0), absolute ethanol, 40 mM EDTA (pH 8.0), 4.2 mM MTT (thiazolyl blue), and 0.2 mL of 16.6 mM PES (phenazine methosulfate). 50 μL extract, 0.3 mL ddH_2_O, 0.6 mL mixture and 50 μL alcohol dehydrogenase (500 U/mL) were then mixed to start the reaction, and the absorbance at 570 nm was measured with the spectrophotometer (TU-1810, PERSEE, Beijing, China) for 5 min. Commercial NADH and NAD^+^ (Meryer Chemical Technology, Shanghai, China) were used to make the standard curves.

### Transcriptomic Analysis

Transcriptomics study was carried out following the method described in previous reports (He et al., 2018; Yang et al., 2018). Briefly, cell culture samples were collected within 24 h fermentation duration with different CH_4_/O_2_ gas mixture ratios. RNA-Seq were performed using the paired-end sequencing technology according to standard Illumina protocols by GENEWIZ, Inc. (Suzhou, China). The quality of RNA-Seq fastq data was checked using FastQC program^1^. Data passing the quality control were imported into CLC Genomics Workbench (version 11.0) for RNA-Seq analysis to get the RPKM values with the reference genome from the MicroScope database^2^. Gene expression normalization, ONE-WAY ANOVA analysis, and hierarchical clustering analysis were conducted using JMP Genomics (version 9.0) to identify differentially expressed genes at different conditions. Genes were determined to be significantly differentially expressed with a selection threshold of *P*-value ≤ 0.01 and log_2_-fold change ≥ 1 (significant induction) or ≤−1 (significant repression). Duplicate samples were used for each condition.

## Results and Discussion

### CH_4_/O_2_ Ratios Affected Cell Growth Especially on Logarithmic Phase

**FIGURE 1:**
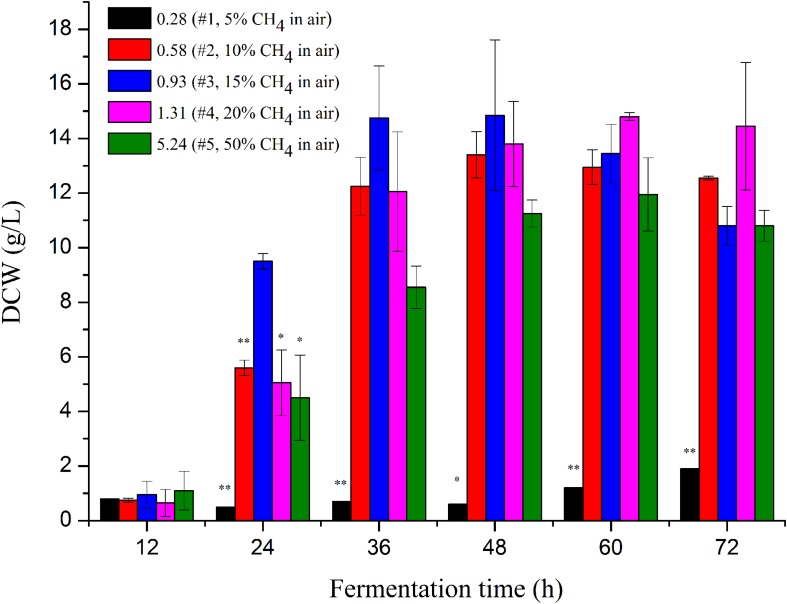
Cell mass of *M. buryatense* 5GB1 in the cultures using different CH_4_/O_2_ ratios of 0.28, 0.58, 0.93, 1.31, and 5.24 at different time points of 12, 24, 36, 48, 60, and 72 h post inoculation, respectively. The * and ** indicated this ratio exhibited significant difference compared to that under the CH_4_/O_2_ ratio of 0.93 with statistical significance of *P* < 0.05 and *P* < 0.01, respectively. DCW, dry cell weight.

Cell growth using various gas mixtures with different CH_4_/O_2_ ratios (from 0.28 to 5.24 mole/mole) were evaluated in 3-L bioreactors in duplicate. Three ratios within the CH_4_ explosion range (5∼15% in air) of 0.28, 0.58, and 0.93 were explored for the first time by using a safety-proof control system in the lab (Fei and Pienkos, 2018). As shown in Figure 1, cells grew the best with a gas mixture ratio of 0.93, which provided the highest specific growth rate of 0.287 h^–1^, followed by 0.219 h^–1^ and 0.198 h^–1^ for gas mixture ratios of 1.31 and 0.58, respectively (Supplementary Table S1).

The highest specific growth rate of *M. buryatense* 5GB1 reported before was 0.231 h^–1^ obtained in cultures with CH_4_/O_2_ of 1.31 (20% CH_4_ in air) (Puri et al., 2015). Our result, for the first time exhibited that the CH_4_/O_2_ ratio of 0.93 is optimal for cell growth of 5GB1, providing a DCW of 15 g/L along with a productivity of 0.3 g/L/h at the time point of 48 h. More importantly, *M. buryatense* 5GB1 exhibited a good state of growth on logarithmic phase, since the biomass at the time points of 24, 36, and 48 h especially the first two time points with the ratio of 0.93 was higher than those with other ratios. It is clear that the growth was significantly limited when cultured under the condition of 5% CH_4_ in the air, which is mainly due to the insufficient carbon source of CH_4_ during cultivation. However, higher CH_4_ supply (e.g., 50% CH_4_ with the ratio of 5.5) did not provide the best cell growth neither (Figure 1 and Supplementary Figure S1). Although cell grew better than that using lower carbon source in cultures under the gas ratio of 0.28, they grew poorly compared with other three conditions with 10∼20% CH_4_, which could be due to limited electron acceptor O_2_ in the gas mixture.

Our findings thus demonstrated the importance of CH_4_/O_2_ ratios in the gas mixtures for balanced cell growth and production cost. The molar ratio of 0.93 was the best among all ratios, which can reach an OD value two-fold of other conditions within 24 h post-inoculation and had the highest OD value within 36∼48 h fermentation time. The shorter time cells took to reach its highest growth and the lower CH_4_ in the gas mixture could mean a higher productivity with less carbon supplied for economic biochemical production.

### Different CH_4_/O_2_ Ratios of Inlet Gas Affected the Accumulation of Key Metabolites and the Level of Reducing Power

Since CH_4_/O_2_ ratios affected cell growth and the ratio of carbon and oxygen was crucial for balanced cell growth, the impact of different CH_4_/O_2_ ratios on the redox and the production of metabolite intermediates were investigated. Several key metabolites and enzymes during CH_4_ oxidation and metabolism were analyzed as shown in Table 1, when *M. buryatense* 5GB1 was cultured with different gas supply ratios. It is obvious that the highest values of pyruvate and pMMO activity were observed in cultures with the gas ratio of 0.93. The higher values of pyruvate amount and pMMO activity were also observed in cultures with the gas ratio of 0.93, which is consistent with the result of growth rate in the same condition. These findings could be due to the fact that some key metabolites were not sufficiently synthesized when either CH_4_ or O_2_ was not supplied sufficiently under other conditions. Higher pyruvate (key precursor for TCA cycle) produced in cultures with gas ratio of 0.93 was corresponding with the higher DCW, which is in good agreement with previous report showing that TCA cycle is essential for the production of both cell mass and reducing power in 5GB1 (Fu et al., 2017).

**TABLE 1:** Quantification of the amount of total protein, pyruvate, NADH, and MMO activity under different CH_4_/O_2_ ratio conditions at time point 24 h post inoculation.

**CH_4_/O_2_ ratio (mol)**	**0.58 (#2)**	**0.93 (#3)**	**1.31 (#4)**	**5.24 (#5)**
Protein (g/L)	1.18 ± 0.07	1.00 ± 0.07	0.75 ± 0.05	0.88 ± 0.00
Pyruvate (mg/g protein)	1.13 ± 0.35	2.91 ± 0.33	1.03 ± 0.26	2.23 ± 0.49
NADH/NAD^+^	0.94 ± 0.10	1.28 ± 0.05	1.61 ± 0.32	0.89 ± 0.02
pMMO (ug/min/g protein)	6.04 ± 1.25	7.0 ± 0.05	4.65 ± 0.40	6.75 ± 1.68

The changes of CH_4_/O_2_ ratios could affect NADH/NAD^+^ ratio, which determined the fluxes of metabolic pathways as well as the transcriptional level of many genes (Zhou et al., 2011). In this work, under the gas ratios of 1.31 and 0.93, the values of NADH/NAD^+^ ratio were higher, and the highest value was 1.61, almost double that under the gas ratio of 5.24 (Table 1). This could be due to the different content of metabolite accumulation and the complicated metabolism in *M. buryatense* 5GB1 with different gas ratios. This process was accompanied by changes of many genes at transcriptional level, so genomic and transcriptomic analyses could help further identify gene and metabolic pathways differentially expressed at different CH_4_/O_2_ ratios.

### Identification of Differentially Expressed Genes at Different Gas Supply Conditions

**FIGURE 2:**
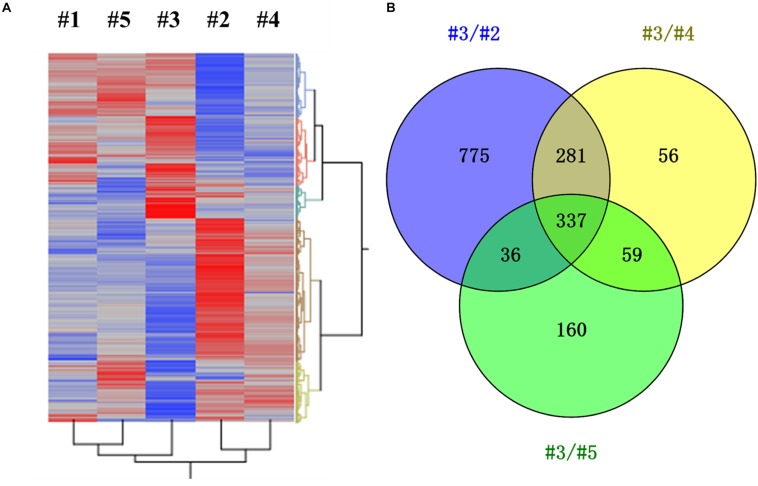
Identification of differentially expressed genes (DEGs) under different gas supply conditions (0.28/#1, 0.58/#2, 0.93/#3, 1.31/#4, 5.24/#5). **(A)** Hierarchical clustering was performed using the LSmean of DEGs, in which significant gene rows were included by comparing any two conditions with at least one *p*-value < 0.01. *X*-axis represented different gas supply conditions and *Y*-axis represented DEGs. **(B)** Venn analysis of DEGs under gas supply ratio of 0.93 (#3) compared with other three conditions of #2, #4, and #5, respectively.

Transcriptomic study was further carried out to investigate the impact of different CH_4_/O_2_ ratios on global gene expression and the underlying mechanism of CH_4_/O_2_ ratio on cell growth and metabolism. As shown in Figure 1, the cell growth under the gas ratio of 0.93 exhibited obvious difference over other gas conditions at 24 h, so we adopted the samples for transcriptome analyses. Since different CH_4_/O_2_ ratios significantly influenced the cell growth and central metabolism, which result in different phenotypes, the relationship of five conditions with different CH_4_/O_2_ ratios were analyzed by hierarchical clustering using significantly differentially expressed genes based on the RNA-Seq result (Figure 2).

The result demonstrated that these samples can be divided into three groups: #3, #2&#4, and #1&#5, which not only indicates that the metabolism of #3 was different from others, but also suggests that the metabolism in cells was different under #2, #4, and #5 conditions despite that they exhibited similar growth (Figure 2A and Supplementary Figure S1). There were total of 1429, 733, and 592 DEGs by comparing #3 with other three conditions of #2, #4, and #5, respectively (Figure 2B and Supplementary Table S2). The DEGs of #3 versus #2, #4 or #5 were analyzed, and the result indicated that 337 common genes were significantly differentially expressed (log_2_fold change ≥ 1, *p*-value ≤ 0.01) with 160 down-regulated and 177 up-regulated ones (Supplementary Table S3). Among these significantly differentially expressed genes, the function of 106 genes was unknown (Supplementary Table S3), which needs further studies to confirm.

Our results also demonstrated that different CH_4_/O_2_ ratios affected both carbon and energy metabolism of *M. buryatense* 5GB1 at transcriptional level (Supplementary Figure S2). Genes up-regulated were mainly involved in signal transduction, membrane transport, cell process, carbohydrate and energy metabolism, while genes down-regulated were majorly involved in metabolism of cofactors and vitamins, nucleotide metabolism and amino acid metabolism. This result indicated that the different phenotypes at condition of 0.93 compared with others were possibly ascribed to the function and expression levels of DEGs involved in carbon and nitrogen utilization, although further investigation is needed.

### Genes Involved in Nitrogen Fixation and Utilization Upregulated to Supply Sufficient Nitrogen for Enhanced Growth

**FIGURE 3:**
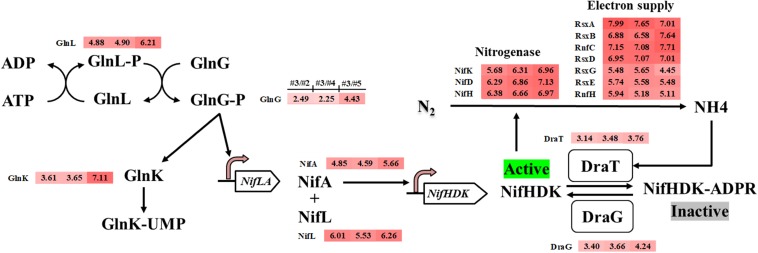
Potential nitrogen fixation regulation system in *M. buryatense* 5GB1 and genes involved in nitrogen fixation regulation upregulated in #3 condition (CH_4_/O_2_ ratio of 0.93) compared with other three conditions of #2, #4, or #5, respectively. The numbers in shadow at right-side of protein name represent the log_2_-based changes of upregulated gene under condition #3 compared to #2, #4, or #5, respectively.

*Methylomicrobium buryatense* 5GB1 has been reported to use nitrate, ammonia, and urea as nitrogen sources (Kaluzhnaya et al., 2001). Some methane-oxidizing bacteria (methanotrophs) are known to be capable of expressing nitrogenase and utilizing N_2_ as the nitrogen source (Auman et al., 2001; Matsen et al., 2013). From the genome annotation and metabolic modeling, *M. buryatense* 5GB1 was also predicted to be able to assimilate N_2_ into ammonia, which can be used as a nitrogen source (de la Torre et al., 2015; Garg et al., 2018). The intriguing discovery from our transcriptomic analysis was that thirty-four nitrogen assimilation related genes were significantly up-regulated in the #3 condition compared with other conditions, including nitrogen fixation structural genes molybdenum nitrogenase genes (*nifHDK*), nitrogenase MoFe maturation genes (*NifPZ*), cofactor biosynthesis genes (*nifBEMNXQTUVY*), ferredoxin genes (*fdxBCD*), as well as genes involved in electron supply (*rnfCH*, *nifF*, *rsxABDEG*), nitrogen fixation regulation (*nifLA, glnGLK*), and post-translational modification (*draTG*) (Figure 3 and Supplementary Table S2).

In *P. stutzeri* A1501, the nitrogen regulatory cascade, AmtB-GlnK-NtrBC, senses the nitrogen signal and controls the expression of *nif*-specific regulatory proteins NifLA, in which the NifA is an activator and the NifL is an anti-activator to control the expression of all other *nif* genes (Lin et al., 2015). In our RNA-Seq result, genes encoding PII protein Glnk, *nif*-specific regulatory protein NifLA, as well as GlnLG homologous to NtrBC were up-regulated under the optimal CH_4_/O_2_ ratio condition #3. The upregulation of nitrogen fixation regulatory genes indicated that the *nif* gene expression in 5GB1 may be tightly regulated at the transcriptional level. Correspondingly, the nitrogenase genes *nifHDK* were up-regulated trigged by this regulatory network under the CH_4_/O_2_ ratio of 0.93, which shown enhanced DCW (Figure 1). These findings in physiological and transcriptional levels may suggest that extra nitrogen source from N_2_ can be assimilated supporting cell growth and metabolic activities after the nitrate source was exhausted, although further study is needed in the future.

While nitrogen fixation is an energy-consuming biological process (Bebout et al., 1993), it is with a high probability to cause the deficiency of biomass yield because of the activation of nitrogen fixation (de la Torre et *al*., 2015). In contrary, better cell growth and higher DCW were achieved along with the higher expression of nitrogen assimilation related genes, which indicated that 5GB1 likely shifted its metabolic flux to overcome the energy barrier for nitrogen fixation. Meanwhile, the nitrogenase post-translational genes (*draTG*), which can switch off or on the nitrogenase activity through adding or removing ADP-ribose group to a specific arginine residue on nitrogenase (Moure et al., 2015), were also up-regulated in response to quickly modulating the activity of nitrogenase when the environmental ammonium concentration was suddenly changed (Masepohl and Hallenbeck, 2010). Additionally, *RsxABCDGE* genes are essential for nitrogen fixation, which are involved in transferring electrons to nitrogenase (Schmehl et al., 1993; Koo et al., 2003). The upregulation of *RsxA*, *RsxB*, *RsxD*, *RsxE*, and *RsxG* (Figure 3) suggested a high nitrogenase activity achieved under #3 condition, which could then facilitate the use of N_2_ as nitrogen source for cell growth. Although higher activity of nitrogenase from #3 condition would promote the nitrogen assimilation, which in turn enhanced the cell growth, the intensive electron cost during nitrogen fixation process could cost cellular energy (Hoffman et al., 2014; Inomura et al., 2018). Unfortunately, research on balancing the N_2_ assimilation and energy supply is still very limited.

It has been reported that higher CH_4_/O_2_ ratio can significantly affect nitrite removal in aerobic methanotrophs, and the highest denitrifying activity correlated with two nitrite reductases (NirK and NirS) was also observed when the molar ratio of CH_4_/O_2_ was 4.4 (Zhu et al., 2017). However, nitrite reductase (NirB) of *M. buryatense* 5GB1 did not show significant differential expression, which may be due to the low CH_4_/O_2_ ratio (0.93) used in the #3 condition.

### The Upregulation of Methane Metabolism Genes Boosted Cell Growth

**FIGURE 4:**
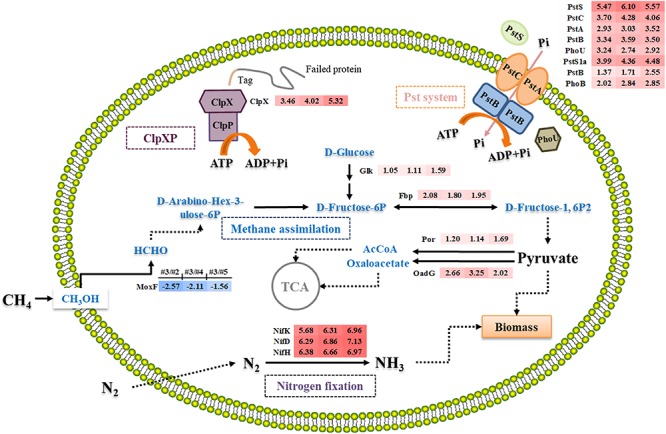
Overview of the differential expression of genes encoding enzymes involving in nitrogen fixation, methane metabolism, ClpX system, and Pst system in *M. buryatense* 5GB1 in condition #3 (0.93) compared with #2 (0.58), #4 (1.31), and #5 (5.24). Dotted arrow indicates multi-step reaction, while solid arrow represents one-step reaction. The numbers in shadow at right-side of protein represent the log_2_-based changes of differentially expressed gene under condition #3 compared to #2, #4, or #5, respectively. The red shadow means upregulation, while the blue shadow means downregulation.

As an aerobic obligate methanotroph, *M. buryatens*e 5GB1 can oxidize CH_4_ to methanol and formate in the beginning before flowing into RuMP and/or serine pathway for assimilations, providing energy for cell growth and metabolic processes at the same time (Trotsenko and Murrell, 2008). It has been reported that the major pathway for pyruvate production in Group I methanotrophic bacteria is the glycolysis pathway (Kalyuzhnaya et al., 2013). Since cell grew better in #3 condition, carbon flux to glycolysis could be enhanced considering the fact that genes related to glycolysis pathway were also upregulated in #3 condition, which is consistent with our result that the expression of *fbp* (MBURv2_60345) was up-regulated greater than two folds (Figure 4).

Fructose-1,6-bisphosphatase is an essential enzyme in glycolysis pathway to hydrolyze D-fructose-1,6-bisphosphate to D-fructose-6-phosphate (Rittmann et al., 2003). Previous study confirmed that Fbp regulated the level of fructose-1,6-bisphosphate, one of the most important intermediates associating with the ATP generation (Li et al., 2013). Besides, Rittmann et al. (2003) investigated the relationship between *fbp* gene and cell growth by deleting it from the chromosome, and discovered that *C. glutamicum* WTΔfbp could not grow as well as the wild-type strain. Although Entner–Doudoroff pathway was suggested to be a potential solution to increase carbon flux for methanotrophic bacteria (Kalyuzhnaya et al., 2015), our result suggests that the enhanced expression of *fbp* gene under the optimal CH_4_/O_2_ ratio can accelerate the conversion of CH_4_ into pyruvate and NADH in *M. buryatense* 5GB1.

### Genes Involved in Key Intermediate Synthesis Upregulated for Enhancing Precursor Supply to TCA Cycle

In both aerobic and anaerobic microorganisms, ATP is an important energy source to sustain microbial growth (Khmelenina et al., 2011). Aerobic microorganisms mainly rely on TCA cycle and oxidative phosphorylation, which provide 95% ATP (Khmelenina et al., 2011). Comparing the transcriptional analysis of #3 with other conditions, two genes (*por* and *oadG*) involved in Acetyl-CoA (AcCoA) and oxaloacetate synthesis were up-regulated (Figure 4), which encode putative oxidoreductase and putative oxaloacetate decarboxylase gamma chain, respectively. Pyruvate was converted to oxaloacetate and AcCoA, which then entered into TCA cycle. The higher production of DCW and NADH observed in #3 condition was therefore consistent with the previous prediction that TCA cycle is responsive for both biomass synthesis and reducing power generation in *M. buryatense* 5GB1 (Fu et al., 2017). This result is also in agreement with pervious study reporting the additional NADH input can support the direct coupling between methane oxidation and methanol oxidation for better cell growth (de la Torre et al., 2015).

### Genes Involved in Phosphate Transport System Upregulated to Maintain Optimal Phosphate Pools for Enhanced Growth

The phosphate uptake systems, which are highly depended on the phosphate transport system (Pst), significantly influence cell growth. Therefore, an efficient Pst system can ensure an enhanced cell mass production (Luz et al., 2012). As shown in Figure 4, six genes (*PstSCAB* and *PstS1a*) belonging to the Pst system were up-regulated under #3 condition comparing with #2, #4, and #5. Additionally, two key regulator genes, *phoB* and *phoU*, encoding phosphate regulon transcriptional regulatory protein (DNA-binding response regulator) and phosphate signaling complex protein (phosphate uptake regulator), respectively, were also upregulated under #3 condition. It was proposed that there was a dynamic equilibrium between active and inactive PstSCAB in response to local internal fluctuations in phosphate concentrations, which was modulated by PhoU (diCenzo et al., 2017). PhoU rapidly responds to elevated phosphate levels by significantly decreasing the phosphate transport of PstSCAB, thereby preventing phosphate toxicity and cell death (diCenzo et al., 2017). It has been known that phosphate is an indispensable compound for cell growth, whereas high concentration phosphate can still be toxic to cell. Therefore, these aforementioned up-regulated genes related to phosphate uptake system may guarantee the rapid response to switch on or off the inorganic phosphate transport system according to cell growth.

Under #3 condition, high cell growth rate probably reflected the high expression level of these genes related to phosphate uptake system that maintains the intracellular Pi pool for optimal growth. Although the mechanism of this system is still elusive and needs more work in the future, our findings provided a new insight into the manipulation of the level of phosphate uptake for enhancing cell growth of methanotrophs, which is possible by controlling CH_4_/O_2_ ratios during the cultivation.

### Genes Involved in ClpX Upregulated to Promote Cell Growth by Controlling ATP Hydrolysis for Energy Supply

As a AAA + protease (ATPase associated with a variety of cellular activities), ClpX hydrolyses ATP to provide energy for targeting the misfolded protein for subsequent degradation during protein synthesis in cell growth process (Keiler et al., 1996; Gottesman et al., 1998; Moore and Sauer, 2007). In addition, the energy produced by hydrolysis of ATP is the main energy source of the ClpXP protease complex in protein degradation. Therefore, upregulated ClpX activated more energy supporting in protein synthesis during cell growth under the #3 condition. Previous studies in *Bacillus subtilis* showed that the cells were damaged readily with the corresponding decrease of the growth rate in the absence of either ClpX or ClpP under stressful environmental conditions (Zhang and Zuber, 2007), which suggests the upregulation of ClpX could be beneficial for protecting cells from damage and promoting the cell growth as what we observed under #3 condition in this study.

## Conclusion

In this work, the impact of different CH_4_/O_2_ ratios on the growth and global gene expression of *M. buryatense* 5GB1 was investigated, and our result indicated that the CH_4_/O_2_ mole ratio of 0.93 is optimal for cell growth, pyruvate accumulation, nutrient utilization and energy supply. Meanwhile, genes related to nitrogen fixation and methane metabolism were significantly up-regulated, which suggest that *M. buryatense* 5GB1 is capable of utilizing N_2_ as the nitrogen source to reduce the production cost. In addition, by controlling CH_4_/O_2_ ratios during the cultivation, the level of phosphate uptake for enhancing cell growth of methanotrophs was improved to maintain an optimal intracellular Pi pool for growth. Our work thus provides novel insights in terms of the influences of CH_4_/O_2_ ratios of gas supply on transcriptional level of key genes in methanotrophic bacteria, and candidate gene targets for future metabolic engineering in this promising industrial methanotrophic microorganism.

## Data Availability Statement

The authors declare that all the data supporting the findings of this study are available within the manuscript and its Supplementary Information files from the corresponding author on request. The RNA-Seq raw data was deposited at Sequence Read Archive (SRA) database with the BioProject accession number PRJNA597286.

## Author Contributions

QF and SY conceived the work, and provided conceptual advice with inputs from all authors. LH designed and performed the experiments, analyzed the data, and wrote the manuscript. YY handled and analyzed the transcriptomic data with help from YC. XY provided conceptual advice. LH, YY, QF, and SY wrote the manuscript, and prepared figures and tables. All authors contributed to data analyses, read, revised and approved the final manuscript.

## Conflict of Interest

The authors declare that the research was conducted in the absence of any commercial or financial relationships that could be construed as a potential conflict of interest.

## Footnotes

1 http://www.bioinformatics.babraham.ac.uk/projects/fastqc/

2 http://www.genoscope.cns.fr/agc/microscope

## Supplementary Material

FIGURE S1 Growth curve of *M. buryatense* 5GB1 under different CH_4_/O_2_ ratios of 0.28, 0.58, 0.93, 1.31, and 5.24, respectively.

FIGURE S2 KEGG enrichment of differentially expressed genes (DEGs) in M. buryatense 5GB1 by comparing 0.93/#3 with 0.58/#2, 1.31/#4, and 5.24/#5. **(a)** Common up-regulated genes in KEGG pathway; **(b)** common down-regulated genes in KEGG pathway.

TABLE S1 Log_2_OD growth data for specific growth rate under different CH_4_/air ratio conditions.

TABLE S2 Differentially expressed genes of #3 (15% CH_4_ in air) compared with #2 (10% CH_4_ in air), #4 (20% CH_4_ in air), and #5 (50% CH_4_ in air).

TABLE S3 Differentially expressed genes of #3 (15% CH_4_ in air) versus #2 (10% CH_4_ in air), #4 (20% CH_4_ in air), and #5 (50% CH_4_ in air). 337 genes were significantly changed (log_2_fold change ≥ 1, *p*-value ≤ 0.01) among these comparisons including 160 down-regulated and 177 up-regulated genes.

## Abbreviations

ANOVA: analysis of variance
CCE: carbon conversion efficiency
CH_4_: methane
ClpX: ATPase and specificity subunit of ClpX-ClpP ATP-dependent serine protease
DCW: dry cell weight
DEGs: differentially expressed genes
DraG: ADP-ribosylhydrolase
DraT: ADP-ribosyltransferase
Fbp: fructose-1,6-bisphosphatase
GHGs: greenhouse gases
Glk: glucokinase
GlnG: response regulator
GlnK: nitrogen assimilation regulatory protein
GlnL: sensory histidine kinase
MoxF: methanol dehydrogenase [cytochrome c] subunit 1
NifA: Nif-specific regulatory protein
NifD: nitrogenase molybdenum-iron protein alpha chain
NifH: nitrogenase iron protein
NifHDK: nitrogenase
NifK: nitrogenase molybdenum iron protein beta chain
NifL: anti-activator of NifA
NMS: nitrate mineral salt
OadG: putative oxaloacetate decarboxylase gamma chain
OD: optical density
PhoB: DNA-binding response regulator in two-component regulatory system with PhoR
PhoU: phosphate uptake regulator
pMMOs: particulate methane monooxygenases
Por: pyruvate-ferredoxin/flavodoxin oxidoreductase
PstA: high-affinity phosphate transport protein membrane subunit
PstB: high-affinity phosphate transporter ATP-binding subunit
PstC: high-affinity phosphate transport protein membrane subunit
PstS: periplasmic phosphate-binding protein
PstS1a: ABC-type phosphate transport system
RBS: ribosome-binding site
RuMP: ribulose-monophosphate.
