# Supplementary figures and images for: Molecular Mechanism Associated With the Impact of Methane/Oxygen Gas Supply Ratios on Cell Growth of Methylomicrobium buryatense 5GB1 Through RNA-Seq
Source: Front Bioeng Biotechnol. 2020 Apr 7;8:263. doi: 10.3389/fbioe.2020.00263 (PMC7154130; doi:10.3389/fbioe.2020.00263)

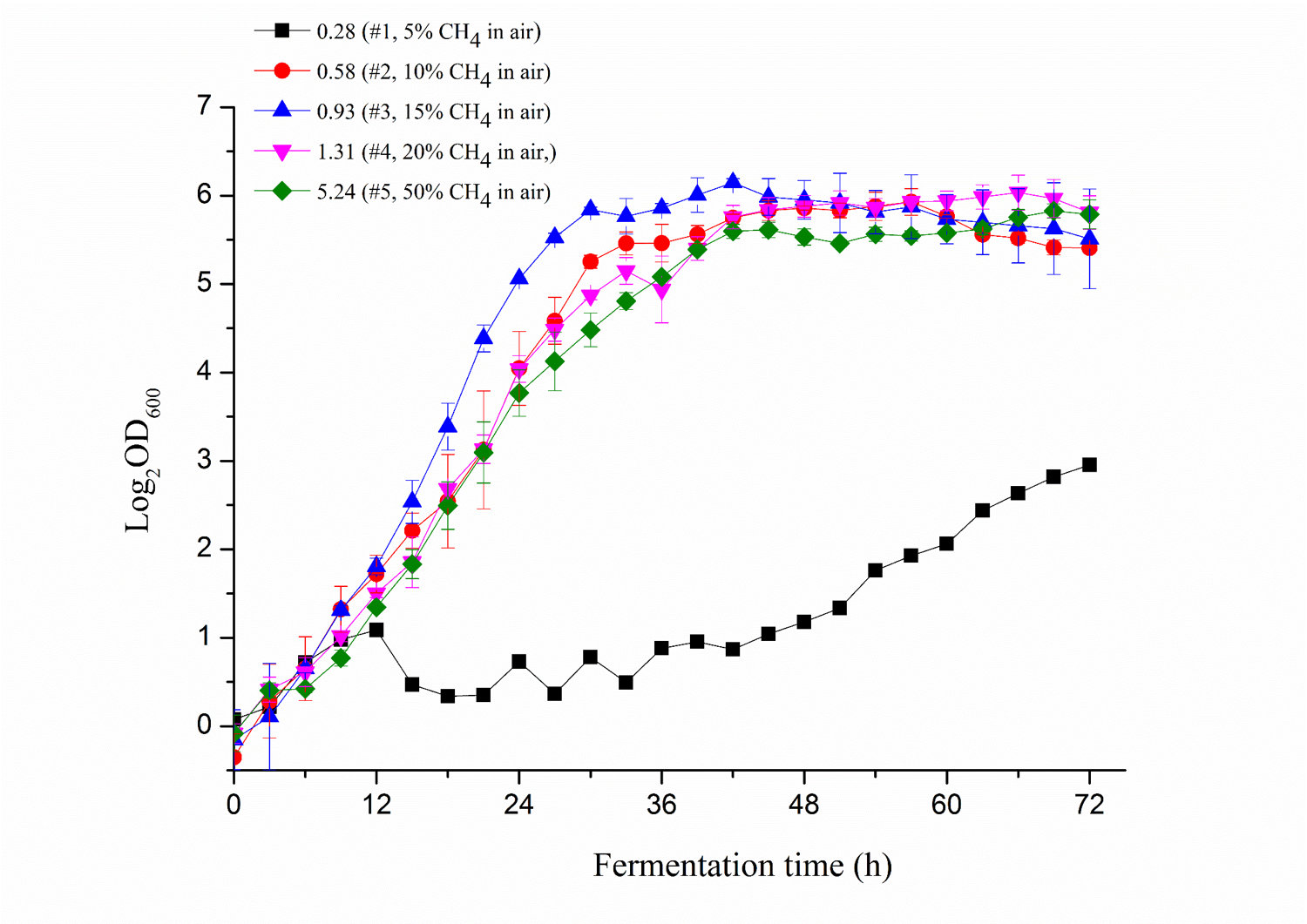

Supplement: FIGURE S1 — Growth curve of M. buryatense 5GB1 under different CH4/O2 ratios of 0.28, 0.58, 0.93, 1.31, and 5.24, respectively. [file Image_1.tif]

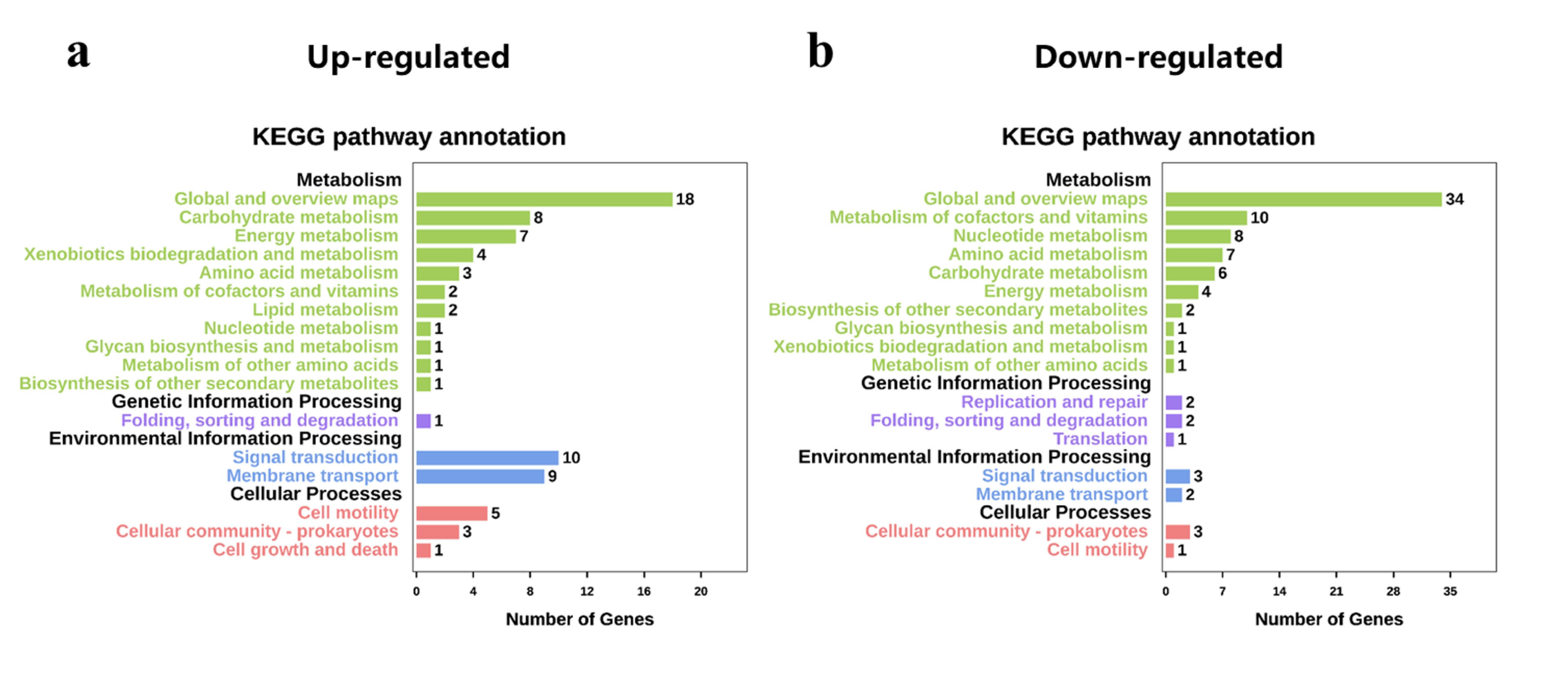

Supplement: FIGURE S2 — KEGG enrichment of differentially expressed genes (DEGs) in M. buryatense 5GB1 by comparing 0.93/#3 with 0.58/#2, 1.31/#4, and 5.24/#5. (a) Common up-regulated genes in KEGG pathway; (b) common down-regulated genes in KEGG pathway. [file Image_2.TIF]
